# Adenosine Deaminase‐Like Gene‐Carried Lentivirus Toolkit for Identification of DNA N^6^‐Methyladenine Origins

**DOI:** 10.1002/advs.202403376

**Published:** 2024-07-18

**Authors:** Ziyu Liang, Shaokun Chen, Yao Li, Weiyi Lai, Hailin Wang

**Affiliations:** ^1^ The State Key Laboratory of Environmental Chemistry and Ecotoxicology Research Center for Eco‐Environmental Sciences Chinese Academy of Sciences Beijing 10085 P. R. China; ^2^ University of Chinese Academy of Sciences Beijing 100049 P. R. China; ^3^ School of Environment and Health Jianghan University Wuhan 430056 China; ^4^ Hangzhou Institute for Advanced Study University of Chinese Academy of Sciences Hangzhou 310024 China

**Keywords:** adenosine deaminase‐like protein, DNA N6‐methyladenine, isotope labeling, UHPLC‐MS/MS

## Abstract

Post‐replicative DNA N^6^‐methyladenine (pr6mdA) can form via bona fide methylase‐catalyzed adenine methylation, playing a pivotal role in embryonic development and other biological processes. Surprisingly, pre‐methylated adenine can be erroneously incorporated into DNA as misincorporated N6‐methyladenine (i6mdA) via DNA polymerase‐mediated replication. Despite pr6mdA and i6mdA sharing identical chemical structures, their biological functions diverge significantly, presenting a substantial challenge in distinguishing between the two. Here, for the first‐time, it is exploited that the adenosine deaminase‐like (Adal) protein and a corresponding activity‐null mutant to construct an Adal lentivirus toolkit. With this newly designed toolkit, both pr6mdA and i6mdA can be identified and quantified simultaneously. The presence of 6mdA in the bone marrow cells of mice is shown, with its levels serving as indicators for growth with age, probably reflecting the cellular stress‐caused changes in RNA decay, nucleotide pool sanitation, and transcription. Collectively, a powerful toolkit to advance understanding of both pr6mdA and i6mdA is demonstrated.

## Graphical abstract

An Adal lentivirus toolkit containing the adenosine deaminase‐like (Adal) protein and a corresponding activity‐null mutant is construct to identify and quantify both post‐replicative DNA N6‐methyladenine (pr6mdA) and misincorporated N6‐methyladenine (i6mdA) simultaneously. With this newly designed toolkit, the level of i6mdA in erythroid cells of mice, which served as potential indicators for growth of age is identified.

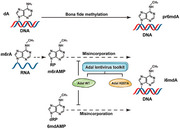

## 1 Introduction

DNA N^6^‐methyladenine (6mdA) is a predominant epigenetic mark in prokaryotes.^[^ ^1^, ^2^ ^]^ In recent years, 6mdA was proposed to exist in many multicellular eukaryotes, including fungi,^[^ ^3^ ^]^ *C. elegans*,^[^ ^4^ ^]^ Drosophila,^[^ ^5^ ^]^ plants,^[^ ^6^, ^7^ ^]^ zebrafish,^[^ ^8^ ^]^ and some mammalian cells.^[^ ^9^, ^10^, ^11^, ^12^ ^]^ This modification has been implicated in the regulation of nucleosome localization,^[^ ^13^ ^]^ organismal development,^[^ ^4^, ^5^, ^8^, ^14^ ^]^ transgenerational inheritance,^[^ ^15^ ^]^ and pathogenesis.^[^ ^16^, ^17^ ^]^ However, 6mdA content is extremely rare in most multicellular eukaryotes, and bacterial DNA carrying with overwhelming 6mdA may contaminate eukaryotic DNA, leading to overestimation of multicellular DNA 6mdA.^[^ ^18^ ^]^ While catalytic methylases are believed to generate 6mdA in multicellular eukaryotes,^[^ ^11^, ^12^, ^19^ ^]^ part of the genomic 6mdA in some mammalian cells originates from the incorporation of pre‐methylated adenosine by DNA polymerase.^[^ ^9^, ^20^ ^]^ This pre‐methylated adenosine likely arises from the decay of RNA N^6^‐methyladenosine (m6rA), leading to the formation of N^6^‐methyl‐2′‐deoxyadenosine triphosphate (6mdATP), which could be directly incorporated into genomic DNA via DNA polymerases.^[^ ^9^ ^]^ Noteworthily, misincorporated i6mdA is generally undetectable in most cells.^[^ ^21^ ^]^ Lyu et al. showed that although it is extremely rare in human glioblastoma, misincorporated i6mdA is a potential hallmark of prognosis.^[^ ^17^ ^]^ Febrimarsa reported that random incorporation of 6mdA into the early embryonic genome may inhibit transcription in *Hydractinia*.^[^ ^22^ ^]^

Given that post‐replicative (pr6mdA) and misincorporated 6mdA (i6mdA) are chemically identical, differentiating between them is challenging. Heavy stable isotope labeled (2′‐deoxy)adenosines have been employed to exclude bacterial DNA contamination^[^ ^23^, ^24^ ^]^ and to trace both pr6mdA and i6mdA.^[^ ^9^, ^23^ ^]^ In certain scenario, i6mdA displays a delayed generation phase, thereby, it can be indirectly discriminated from pr6mdA using heavy stable isotope tracing.^[^ ^20^, ^21^ ^]^ We propose that, by setting up a metabolic barrier against the formation of 6mdATP, the production of i6mdA could be selectively prevented, allowing for the simultaneous distinction and quantification of both i6mdA and pr6mdA.

Adenosine deaminase‐like (Adal) protein is an ideal candidate for establishing this metabolic barrier. Adal, a member of the adenyl deaminase family, contains conserved catalytic residues earlier observed in adenosine deaminase (ADA) and adenosine deaminase‐related growth factors (ADGF).^[^ ^25^ ^]^ Notably, Adal preferentially hydrolyzes N^6^‐methyl‐adenosine 5′‐monophosphate (m6rAMP) and N^6^‐methyl‐2′‐deoxyadenosine 5′‐monophosphate (6mdAMP) without affecting adenosine and adenosine 5′‐monophosphate (AMP).^[^ ^26^ ^]^ This specificity suggests that Adal activity could prevent the formation of 6mdATP by hydrolyzing its precursors, thereby reducing misincorporated i6mdA. Chen et al. observed that m6rAMP is accumulated in Adal ‐knocked out Arabidopsis thaliana.^[^ ^27^ ^]^ We showed that the depletion of Adal in mammalian cells elevates free 6mdA and i6mdA levels.^[^ ^21^ ^]^

Regarding that m6rAMP and 6mdAMP are indispensable intermediates to form 6mdATP via purine salvage pathway, one barrier to remove both m6rAMP and 6mdAMP are highly desirable. Adal can eliminate 6mdATP by hydrolyzing both m6rAMP and 6mdAMP and consequently eliminate misincorporated DNA i6mdA, therefore, Adal will be ideal metabolic barrier to eliminate DNA i6mdA. Based on this inference, we for the first time designed a novel Adal lentivirus toolkit to identify and quantify both pr6mdA and i6mdA. This toolkit represents a significant advancement in our ability to distinguish these two forms of 6mdA, which is crucial for an accurate understanding of their distinct biological functions.

## 2 Results and Discussion

### 2.1 Design of Deaminase‐Null Adal Mutant

**Figure 1:**
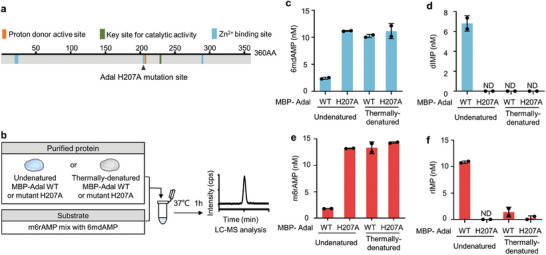
Selection of *Adal* protein mutant as a control for toolkit identifying 6mdA origins. a) The key sites of mouse Adal (UniProt ID: Q80SY6). Adal H207A mutation site is marked with black triangle symbol. b) Flow diagram of the catalytic activity assay for purified MBP‐ Adal WT and mutant H207A. c–f) Quantification of substrates (6mdAMP c) and m6AMP e)) and products (dIMP d) and rIMP f)) upon deamination reaction mediated by purified MBP‐ Adal WT and mutant H207A. *n* = 2 independent biological replicates. ND: not detectable.

Previous studies have pointed out that ADAL and ADA from various species possess highly conserved domains, which include four Zn^2+^‐binding amino acid residues.^[^ ^25^, ^28^ ^]^ Alterations to these Zn^2+^‐binding amino acid residues in mouse ADA have been shown to result in the loss of enzyme activity.^[^ ^29^, ^30^ ^]^ Given the pivotal role of the Zn^2+^‐binding residues played in ADA enzyme activity, we postulated that similar alterations could lead to a reduction or elimination of its enzyme activity. We aligned eukaryotic ADAL protein sequences (http://www.uniprot.org) with those of human and murine ADA, focusing on the amino acid residues associated with the Zn^2+^ binding in mouse Adal (**Figure** **1**a; Figure S1a, Supporting Information). Subsequently, we introduced a mutation at one of the Zn^2+^‐binding amino acid residues, changing Histidine 207 to Alanine (H207A) (Figure S1b, Supporting Information). To assess the in vitro catalytic activity (Figure 1b), we purified both N‐terminally MBP‐tagged wild‐type Adal and the mutant H207A proteins (Figure S1c, Supporting Information).

Ultra high‐performance liquid chromatography coupled with tandem mass spectrometry (UHPLC‐MS/MS) was employed to quantify the conversion rates of 6mdAMP and m6rAMP into dIMP and IMP, respectively. To improve the detection sensitivity, both the substrates (6mdAMP and m6rAMP) and the products (dIMP and IMP) were analyzed in their dephosphorylated forms. The data were then recalibrated to account for their phosphorylated states. As a negative control, we observed that thermally denatured proteins did not consume any substrates nor produce products. Conversely, As a positive control, in a reaction system of 50 µL, wild‐type Adal efficiently catalyzed the deamination of 11.5 nM m6rAMP and 7.8 nM 6mdAMP, yielding 9.5 nM IMP and 6.8 nM dIMP, respectively (Figure 1c–f; Figure S2, Supporting Information). Importantly, the H207A mutant did not convert any substrates into products, indicating a complete loss of hydrolase activity due to the mutation of the Zn^2+^‐binding residue H207 to alanine.

### 2.2 Construction of Adal Gene‐Carried Lentivirus Toolkit

**Scheme 1:**
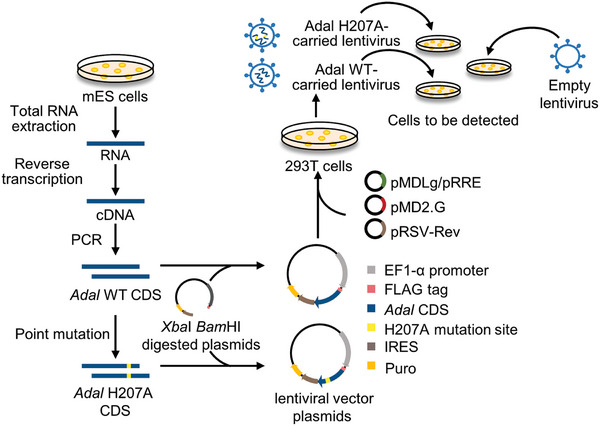
Construction of Adal lentivirus toolkit. We amplified Adal WT CDS from cDNA reverse transcribed from total RNA of mES cells, and used point mutation to gain Adal H207A CDS. Then, we inserted CDS of Adal WT and mutant H207A into the pLVX‐EF1α‐IRES‐puro lentivirus vector plasmids. After transfecting lentivirus vectors plasmids into HEK‐293T cells, we obtained the Adal lentivirus toolkit for the detection of two types of 6mdA. Empty lentivirus served as a negative control.

**Figure 2:**
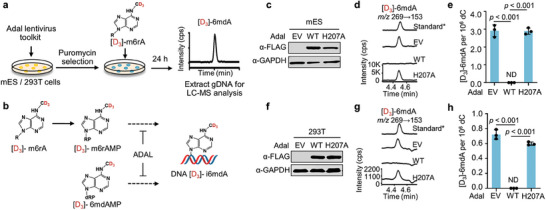
Complete elimination of DNA i6mdA misincorporation by Adal lentivirus toolkit. a) Flow diagram of assessing the 6mdA misincorporation by Adal lentivirus toolkit. b) Schematic illustration of Adal eliminating isotope‐labeled i6mdA. c) The western blot analysis of overexpressed Adal in mES cells. d,e) UHPLC‐MS/MS chromatograms d) and quantification e) of [D_3_]−6mdA in mES cells that overexpressed Adal. All mES cells were treated with 1 µM [D_3_]‐m6rA for 24 h. f) The western blot analysis of overexpressed Adal in HEK‐293T. g,h) UHPLC‐MS/MS chromatograms g) and quantification h) of [D_3_]−6mdA in Adal ‐overexpressed HEK‐293T cells. All HEK‐293T cells were treated with 5 µM [D_3_]‐m6rA for 24 h. gDNA: genomic DNA. EV: empty vector. WT: wild‐type. Standard*: non‐labeled Standard. Error bars are S.D. (*n* = 3 independent biological replicates). ND: not detectable.

To differentiate between the two types of 6mdA, we inserted the coding sequence (CDS) fragments of mouse wild‐type *Adal* and mutant H207A into the pLVX‐EF1α‐FLAG‐IRES‐Puro vector, respectively. The verification of the insertions was confirmed by Sanger sequencing (Figure S3, Supporting Information). Subsequently, the constructed lentiviral plasmids were transfected into HEK‐293T cells. Based on above experiments, we have developed a comprehensive lentivirus toolkit that encompasses both the mouse wild‐type Adal and the H207A mutant (**Scheme** **1**). An Empty lentivirus (EV) was served as a negative control for further research. We initially applied this toolkit to mouse embryonic stem (mES) cells and human embryonic kidney HEK‐293T cells to establish model systems. After successful infection with lentivirus, polyclonal stable cell lines were generated using puromycin selection (**Figure** **2**a,b).

### 2.3 Complete Elimination of i6mdA by Overexpressed Adal

Treatment with the modified nucleoside m6rA can result in the misincorporation of DNA i6mdA into cellular DNA.^[^ ^20^, ^21^ ^]^ Consequently, we treated Adal‐overexpressed mES and HEK‐293T cells with [D_3_]‐m6rA to induce i6mdA in the form of [D_3_]−6mdA (Figure 2a,b). Utilizing the highly sensitive UHPLC‐MS/MS assay, we detected [D_3_]−6mdA at a level of about 2.91 per 10^6^ dC in mES cells transduced with either empty lentivirus vector or the H207A mutant (Figure 2c–e). In HEK‐293T cells transduced with the same vectors, the levels of [D_3_]−6mdA were 0.72 and 0.59 per 10^6^ dC, respectively (Figure 2f–h). These findings concur with those reported in previous studies.^[^ ^9^, ^21^, ^23^ ^]^ Interestingly, we scarcely detected any signals of DNA [D_3_]−6mdA in mES and HEK‐293T cells that overexpressed wild‐type Adal (Figure 2d,e,g,h). These data suggest that overexpression of wild‐type Adal eliminates all i6mdA in mammalian cells, while the H207A mutant overexpression does preserve any of the generated DNA 6mdA.

### 2.4 Preservation of pr6mdA in Adal‐Overexpressed Cells

**Figure 3:**
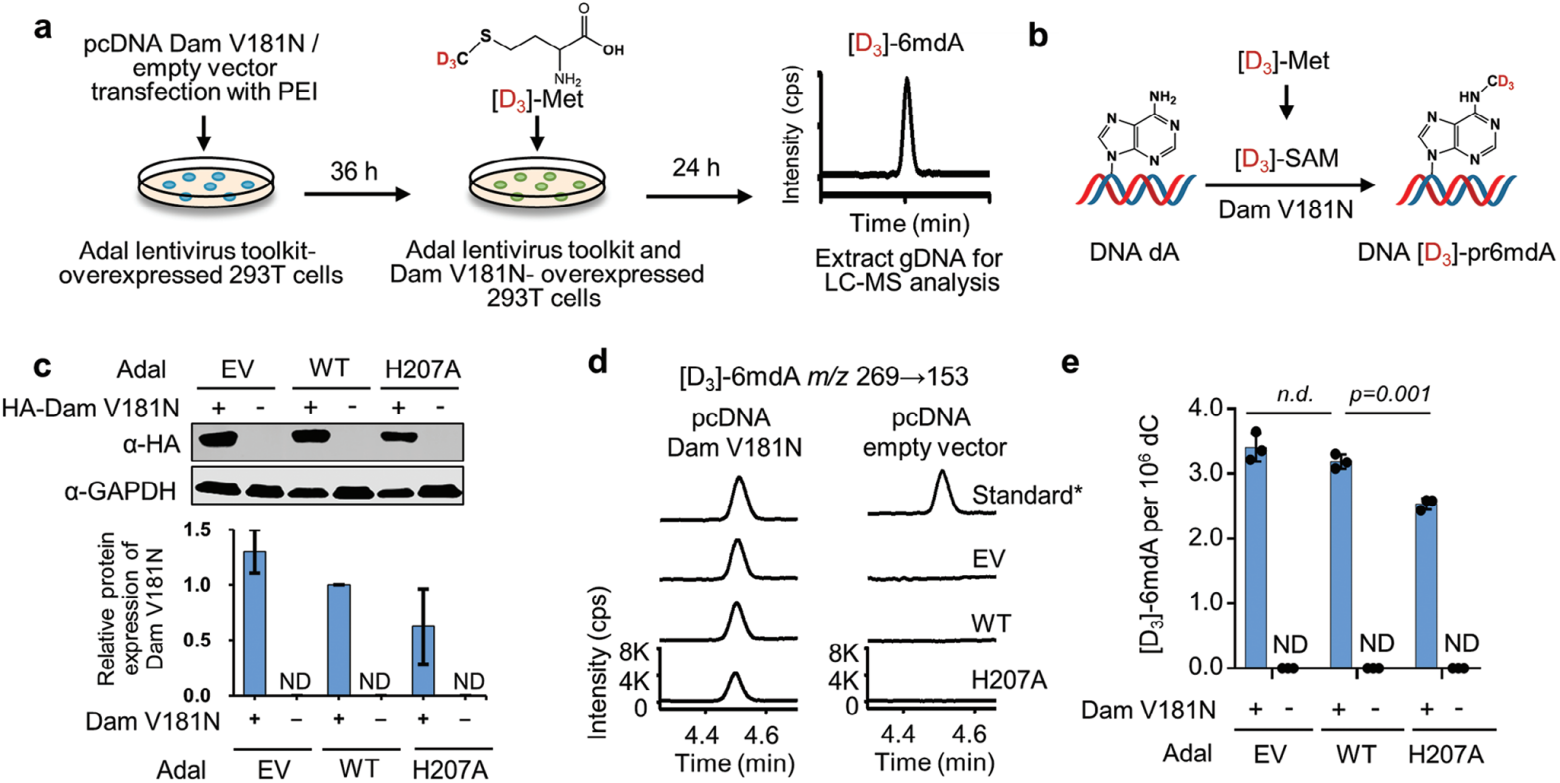
Methylase‐generated pr6mdA can be retained as assessed by Adal lentivirus toolkit. a) Flow diagram of assessing pr6mdA by Adal lentivirus toolkit. b) Schematic illustration of isotope‐labeled pr6mdA generation by activity‐reduced methylase Dam V181N. c) The western blot analysis and quantification of HA‐tagged Dam V181N overexpression in Adal lentivirus toolkit‐overexpressed HEK‐293T cells. Protein relative density was normalized to Adal WT. d,e) UHPLC‐MS/MS chromatograms d) and quantification e) of [D_3_]−6mdA in HEK‐293T cells that simultaneous overexpressed Dam V181N and Adal. All HEK‐293T cells were treated with 30 µg mL^−1^ [D_3_]‐L‐methionine ([D_3_]‐Met). gDNA: genomic DNA. EV: empty vector. WT: wild‐type. Standard*: non‐labeled Standard. Error bars are S.D. (*n* = 3 independent biological replicates). ND: not detectable.

Next, we sought to determine if the Adal lentivirus toolkit influences the production of pr6mdA (**Figure** **3**a,b). Thus, we transfected pcDNA‐Dam V181N‐3 × HA plasmids or pcDNA empty vectors into HEK‐293T cells that overexpressed Adal lentivirus toolkit. Dam V181N is a *E. coli* adenine methylase Dam mutant capable of catalyzing the synthesis of pr6mdA but with reduced activity. By employing the *E. coli* Dam V181N mutant instead of the wild‐type enzyme, we aimed to prevent cross‐cellular contamination with DNA 6mdA due to extremely high activity of the wild‐type Dam. Western blot analysis confirmed the successful overexpression of both the Adal and Dam V181N proteins in HEK‐293T cells (Figures 2f and 3c). The cells were treated with [D_3_]‐methionine ([D_3_]‐Met) to indicate the generation of pr6mdA ([D_3_]−6mdA) by Dam. The UHPLC‐MS/MS assay showed that in HEK‐293T cells transfected with Dam V181N, the levels of [D_3_]−6mdA were comparable in each group, as 3.41, 3.19, and 2.53 [D_3_]−6mdA per 10^6^ dC were detected for EV, Adal WT and H207A. In contrast, no isotope‐labeled 6mdA was undetectable in pcDNA empty vectors transfected cells (Figure 3d,e). Although there is a subtle difference in Dam V181N overexpression, the levels of pr6mdA generated by overexpressed Dam V181N are not statistically significant diminished by wild‐type Adal using Adal toolkit (Figure 3d,e), therefore, it does not affect the elucidation of the observed pr6mdA using Adal toolkit, even for quantification. In contrast, all misincorporated i6mdA could be eliminated by wild‐type Adal using Adal toolkit. These results collectively indicated that Adal toolkit can efficiently distinguish between pr6mdA and i6mdA(Figure S4, Supporting Information).

### 2.5 Identification and Quantification of i6mdA in Cultured Cells

**Figure 4:**
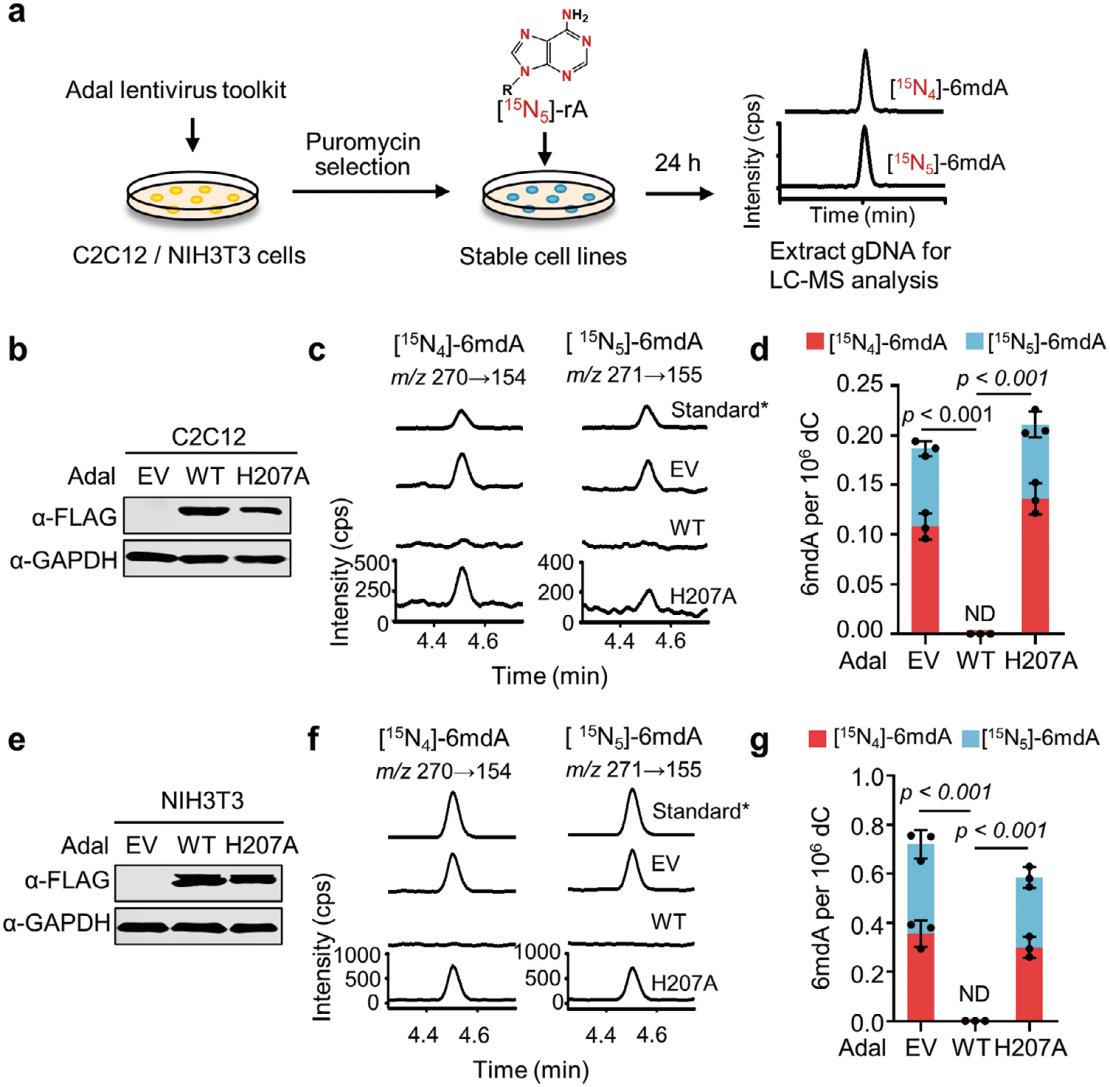
Identification and quantification of i6mdA in C2C12 and NIH3T3 cells by Adal lentivirus toolkit. a) Flow diagram of identifying the origin of 6mdA in C2C12 and NIH3T3 cells by Adal lentivirus toolkit. b) The western blot analysis of overexpressed Adal in C2C12 cells. c,d) UHPLC‐MS/MS chromatograms c) and quantification d) of labeled 6mdA in Adal‐overexpressed C2C12 cells. All C2C12 cells were treated with 20 µM [^15^N_5_]‐rA for 36 h. e) The western blot analysis of overexpressed Adal in NIH3T3 cells. f,g) UHPLC‐MS/MS chromatograms f) and quantification g) of labeled 6mdA in Adal‐overexpressed NIH3T3 cells. All NIH3T3 cells were treated with 20 µM [^15^N_5_]‐rA for 36 h. gDNA: genomic DNA. EV: empty vector. WT: wild‐type. Standard*: non labeled Standard. Error bars are S.D. (*n* = 3 independent biological replicates). ND: not detectable.

Previous studies have reported that the presence of rare i6mdA in mouse myoblast C2C12 cells and mouse embryonic fibroblast NIH3T3 cells.^[^ ^20^, ^21^ ^]^ We next examined this observation with Adal lentivirus toolkit (**Figure** **4**a). Western blotting confirmed successful overexpression of both wild‐type Adal and the H207A mutant (Figure 4b,e). To eliminate any potential bacterial DNA 6mdA contamination, we treated both C2C12 and NIH3T3 cells with isotope‐labeled [^15^N_5_]‐rA. Consistent with our group's earlier report,^[^ ^21^ ^]^ the cells labeled with [^15^N_5_]‐rA generated two variants of labeled 6mdA: [^15^N_4_]−6mdA and [^15^N_5_]−6mdA. In C2C12 cells overexpressing either the empty vector or the H207A mutant, the abundances of the isotope‐labeled 6mdA were 0.19 and 0.21 per 10^6^ dC, respectively (Figure 4c,d). NIH3T3 cells exhibited a higher incorporation of i6mdA, with measurements of 0.72 labeled‐6mdA per 10^6^ dC overexpressing the empty vector and 0.59 isotope‐labeled 6mdA per 10^6^ dC in those overexpressing the mutant H207A (Figure 4f,g). Importantly, isotope‐labeled 6mdA was undetectable in C2C12 and NIH3T3 cells overexpressing wild‐type Adal (Figure 4c,d,f,g). The ratios of isotope‐labeled rA to total rA were found to be 41.9–63.4% in the C2C12 cell groups and 51.1–60.0% in the NIH3T3 cell groups (Figure S5a,b, Supporting Information). Collectively, these findings corroborated that the detected 6mdA in C2C12 and NIH3T3 cells is extremely rare and exclusively originates from the salvage pathway.

### 2.6 The Elevation of i6mdA Abundance During Erythroblast Differentiation

**Figure 5:**
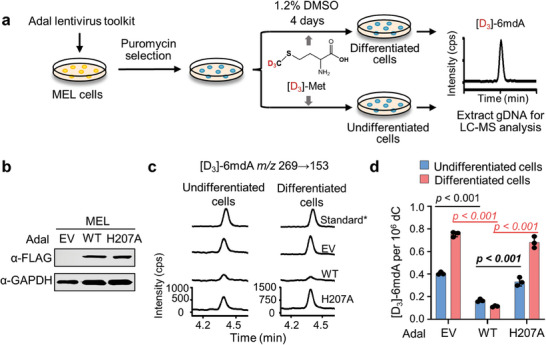
MEL cell differentiation promotes the misincorporation of i6mdA. a) Flow diagram of identifying the type of 6mdA in MEL cells with Adal lentivirus toolkit. b) The western blot analysis of overexpressed Adal in MEL cells. c,d) UHPLC‐MS/MS chromatograms c) and quantification d) of [D_3_]−6mdA in Adal‐overexpressed MEL cells. All MEL cells were treated by 30 µg mL^−1^ [D_3_]‐L‐methionine ([D_3_]‐Met) during differentiation experiment. EV: empty vector. WT: wild‐type. Error bars are S.D. (*n* = 3 independent biological replicates).

**Figure 6:**
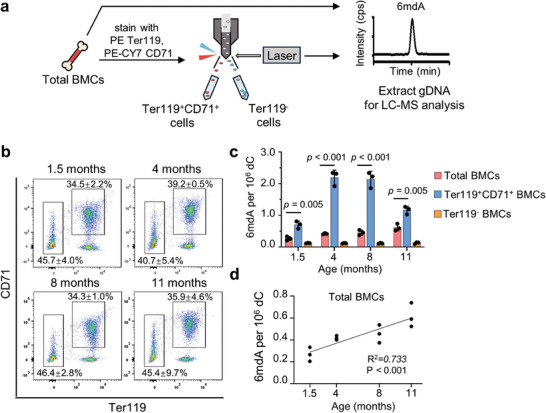
DNA 6mdA in mouse BMCs and its correlation with age. a) Flow diagram of isolating BMCs from C57BL/6J mice of different ages. b) Flow cytometry sorting erythroblasts using antibodies against Ter119, CD71 of BMCs isolated from C57BL/6J male mice of different ages. c) UHPLC‐MS/MS quantification of 6mdA of total BMCs, Ter119+CD71+ BMCs, and Ter119‐ BMCs separated from C57BL/6J male mice of different ages. Error bars are S.D. (*n* = 3 independent biological replicates). d) The relationship between genomic 6mdA level and age in mouse total BMCs. A simple linear regression analysis was performed. Linear regression line, R‐squared, and *p*‐value were shown.

Erythropoiesis is a tightly regulated process by which mature red blood cells are derived from hematopoietic stem cells, including early‐stage erythropoiesis and terminal erythroid differentiation.^[^ ^31^ ^]^ The mouse erythroleukemia (MEL) cell line, established by Friend et al. in early 1970s,^[^ ^32^ ^]^ has been widely used as an in vitro model for studying hematopoietic cell differentiation. Cultured MEL cells closely resemble colony‐forming units erythroid (CFU‐E), maintaining the characteristics of late erythroid progenitor cells. MEL cells can be induced to differentiation by dimethylsulfoxide (DMSO).^[^ ^33^ ^]^ We unexpectedly noted a remarkably increase of 6mdA level after DMSO treatment. To investigate the origin of this increase, we performed lentivirus transduction on MEL cells to achieve stable overexpression of the Adal lentivirus toolkit (**Figure** **5**b). Subsequently, we induced differentiation using DMSO and utilized [D_3_]‐Met to label newly synthesized [D_3_]−6mdA, thereby tracking it throughout the differentiation process (Figure 5a). In undifferentiated MEL cells transduced with either empty lentivirus vehicles or those overexpressing mutant H207A, we detected [D_3_]−6mdA levels of 0.40 and 0.33 per 10^6^ dC, respectively. Notably, a significant lower [D_3_]−6mdA level was observed in cells overexpressing wild‐type Adal (0.16 6mdA per 10^6^ dC) (**Figure** **6**c,d). Similar pattern was also observed in DMSO‐induced differentiated MEL cells (Figure 5c,d). Furthermore, by calculating the percentage increase of [D_3_]−6mdA levels during cell differentiation, we discerned that [D_3_]−6mdA levels roughly doubled in MEL cells treated with either the empty lentivirus vehicle or the H207A mutant. Meanwhile, we verified that [D_3_]‐Met utilization was consistent across all groups, as by similar [D_3_]‐m6rA ratio (ranging from 72.6% to 79.3%) (Figure S5c, Supporting Information). Collectively, both undifferentiated and differentiated MEL cells experience misincorporation of DNA i6mdA, with differentiation further amplifying this misincorporation. Noteworthily, overexpression of wild‐type Adal did not completely diminish the isotope‐labeled 6mdA, probably hinting at the potential presence of minor pr6mdA in MEL cells.

### 2.7 The Abundance of 6mdA in Bone Marrow Cells Potentially Marks Growth with Age

The identification of i6mdA in MEL cells led us to hypothesize that i6mdA could also be present in vivo within blood cells and play a biological role. For this purpose, we collected bone marrow cells (BMCs) from C57BL/6J male mice across various age groups. We then sorted erythroblasts (Ter119+CD71+ cells)^[^ ^34^ ^]^ and Ter119‐ cells using flow cytometry (**Figure** **6**a,b) for precise 6mdA quantification. Utilizing UHPLC‐MS/MS, we found the overall abundance of 6mdA in BMCs showed an age‐dependent increase: levels of 0.27, 0.41, 0.46, and 0.62 6mdA per 10^6^ dC were detected in mice with increasing age, respectively (Figure 6c). As analyzed by simple linear regression, the 6mdA level in total BMCs of mice is significantly positively correlated with age (Figure 6d). A comparable elevation pattern of 6mdA, similar to that observed during MEL cell differentiation, was detected: a significant higher level of 6mdA (1.9–5.3 folds) was observed in bone marrow‐derived erythroblasts compared to less differentiated erythroid cells (Ter119‐ cells), which remained constant across all age brackets (Figure 6c). Based on these findings, it can be speculated that the observed 6mdA in bone marrow‐derived erythroid cells is likely the result of misincorporation. Moreover, we observed that 6mdA in isolated erythroblasts is higher in adult mice (1.16–2.19 per 10^6^ dC, 4 and 8 months) compared to their juvenile counterparts (0.70 per 10^6^ dC, 1.5 months) with a decrease in the elder group (11 months). Collectively, our results indicate that the abundance of 6mdA in BMCs could serve as a potential mark for growth with age.

Previous studies have reported that MTH1 can hydrolyze 6mdATP and m6rATP to generate 6mdAMP and m6rAMP, respectively,^[^ ^35^ ^]^ while Adal is recognized for hydrolyzing both m6rAMP and 6mdAMP from the nucleotide pool.^[^ ^26^, ^27^ ^]^ Moreover, our previous work showed that knockdown of Ak1 in mammalian cells effectively prevents 6mdA incorporation.^[^ ^21^ ^]^ Nevertheless, these mechanisms collectively safeguard against the erroneous incorporation of 6mdA into DNA. Drawing inspiration from these mechanisms, we developed an Adal lentivirus toolkit to identify and quantify misincorporated i6mdA. We have successfully overexpressed wild‐type Adal and its inactive H207A mutant in NIH3T3 and C2C12 cell lines using synthesized lentiviral vectors, respectively. Our observations revealed that overexpression of the wild‐type Adal eliminates the 6mdA, while the H207A mutant does not produce the same outcome. These results collectively suggest that the detectable 6mdA in NIH3T3, and C2C12 cell lines predominantly consists of misincorporated i6mdA. Using our Adal lentivirus toolkit, we found that differentiation of MEL cells enhances the production of i6mdA.

It is particularly noteworthy that the levels of isotope‐labeled 6mdA, deposited by overexpressed 6mdA methylase Dam V181N remained constant in HEK‐293T cells that were co‐overexpressing the wild‐type Adal. This suggests that the toolkit selectively targets and eliminates the generated i6mdA without affecting adenosine methylase‐deposited pr6mdA, thereby estimating the levels of bona fide DNA pr6mdA in mammalian cells. It expects that the established lentivirus toolkit can be applied to detect and quantify both i6mdA and pr6mdA not only in vitro cultured cells but also in ex vivo cells. Furthermore, there is potential for using this toolkit to transfect model organisms for the in vivo investigation of i6mdA and pr6mdA.

As organisms age, there's a progressive decline in biological functions across molecular, cellular, tissue, and organismal levels.^[^ ^36^ ^]^ In humans, advancing age is a significant risk factor for various diseases, including neurodegeneration, diabetes, and cancer. A few epigenetic marks, like histone modifications, and DNA methylation, have been linked to aging in a sequencing dependent manner.^[^ ^37^, ^38^, ^39^ ^]^ In this work, we've noted an increased level of 6mdA in mature erythroid cells in both in vitro cultured cells and in vivo mice models. As tracked using our specially developed Adal lentivirus toolkit, we showed that the differentiation of MEL cell promotes the generation of i6mdA. Additionally, we've identified the presence of 6mdA within total BMCs and erythroblasts of mice. Those 6mdAs might be largely attributed to the DNA replication‐dependent misincorporation.

Crucially, there's a significant positive correlation between the abundance of 6mdA in total BMCs and the age of mice. As individual mice age, stresses factors such as cell death become more prevalent. Our recent work has shown that there's a link between the levels of i6mdA and cellular stresses caused by cell death.^[^ ^17^ ^]^ Therefore, the 6mdA levels we are seeing in total BMCs could be indicative of stress associated with age‐dependent growth. This stress can lead to altered RNA m6rA decay and less effective cleanup of the nucleotide pool. As RNA m6rA decay accelerates, premethylated m6rA nucleoside/nucleotide may be accumulated. Moreover, the mechanisms that cleanse the nucleotide pool of these premethylated m6rA nucleoside/nucleotide^[^ ^21^ ^]^ may become less efficient with age. Consequently, these growth‐associated stresses may result in a rise in i6mdA level within BMCs. Furthermore, a preceding study has documented that i6mdA, present in the early embryos of the cnidarian *Hydractinia symbiolongicarpus*, exerts a repressive effect on transcription.^[^ ^22^ ^]^

Above insights have led us to hypothesize that i6mdA within BMCs may serve as an indicator for growth with age through four potential mechanisms: 1) increased cellular stresses, 2) accelerated decay of RNA m6rA, 3) compromised sanitation of the nucleotide pool, and 4) disruption of normal transcription processes. Unlike other epigenetic marks, the misincorporated 6mdA mark is independent of DNA sequence. Thus, measuring i6mdA abundance could directly serve as an assessment tool for growth with age.

## 3 Conclusion

In summary, while the precise function of 6mdA in eukaryotes continues to be debated, our findings offer new insights into its potential role. We for the first time utilized the adenosine deaminase‐like (Adal) protein and an activity‐null mutant to develop an Adal lentivirus toolkit. This novel toolkit enables the simultaneous identification and quantification of both pr6mdA and i6mdA. In addition, our study suggests that 6mdA may act as a DNA sequence‐independent mark for growth with age. Future research should aim to clarify this modification's role at both cellular and organismal levels. Our innovative methodology holds promise for broad application in such investigations.

## Conflict of Interest

The authors declare no conflict of interest.

## Supporting information

Supporting Information

## Data Availability

The data that support the findings of this study are available from the corresponding author upon reasonable request.

## References

[1] B. F. Vanyushin , A. N. Belozersky , N. A. Kokurina , D. X. Kadirova , Nature 1968, 218, 1066. 5656625 10.1038/2181066a0

[2] G. Fang , D. Munera , D. I. Friedman , A. Mandlik , M. C. Chao , O. Banerjee , Z. Feng , B. Losic , M. C. Mahajan , O. J. Jabado , G. Deikus , T. A. Clark , K. Luong , I. A. Murray , B. M. Davis , A. Keren‐Paz , A. Chess , R. J. Roberts , J. Korlach , S. W. Turner , V. Kumar , M. K. Waldor , E. E. Schadt , Nat. Biotechnol. 2012, 30, 1232. 23138224 10.1038/nbt.2432 PMC3879109

[3] S. J. Mondo , R. O. Dannebaum , R. C. Kuo , K. B. Louie , A. J. Bewick , K. LaButti , S. Haridas , A. Kuo , A. Salamov , S. R. Ahrendt , R. Lau , B. P. Bowen , A. Lipzen , W. Sullivan , B. B. Andreopoulos , A. Clum , E. Lindquist , C. Daum , T. R. Northen , G. Kunde‐Ramamoorthy , R. J. Schmitz , A. Gryganskyi , D. Culley , J. Magnuson , T. Y. James , M. A. O'Malley , J. E. Stajich , J. W. Spatafora , A. Visel , I. V. Grigoriev , Nat. Genet. 2017, 49, 964. 28481340 10.1038/ng.3859

[4] E. L. Greer , M. A. Blanco , L. Gu , E. Sendinc , J. Liu , D. Aristizábal‐Corrales , C. H. Hsu , L. Aravind , C. He , Y. Shi , Cell 2015, 161, 868. 25936839 10.1016/j.cell.2015.04.005 PMC4427530

[5] G. Zhang , H. Huang , D. Liu , Y. Cheng , X. Liu , W. Zhang , R. Yin , D. Zhang , P. Zhang , J. Liu , C. Li , B. Liu , Y. Luo , Y. Zhu , N. Zhang , S. He , C. He , H. Wang , D. Chen , Cell 2015, 161, 893. 25936838 10.1016/j.cell.2015.04.018

[6] C. Zhou , C. Wang , H. Liu , Q. Zhou , Q. Liu , Y. Guo , T. Peng , J. Song , J. Zhang , L. Chen , Y. Zhao , Z. Zeng , D. X. Zhou , Nat Plants 2018, 4, 554. 30061746 10.1038/s41477-018-0214-x

[7] Z. Liang , L. Shen , X. Cui , S. Bao , Y. Geng , G. Yu , F. Liang , S. Xie , T. Lu , X. Gu , H. Yu , Dev. Cell 2018, 45, 406. 29656930 10.1016/j.devcel.2018.03.012

[8] J. Liu , Y. Zhu , G. Z. Luo , X. Wang , Y. Yue , X. Wang , X. Zong , K. Chen , H. Yin , Y. Fu , D. Han , Y. Wang , D. Chen , C. He , Nat. Commun. 2016, 7, 13052. 27713410 10.1038/ncomms13052 PMC5059759

[9] X. Liu , W. Lai , Y. Li , S. Chen , B. Liu , N. Zhang , J. Mo , C. Lyu , J. Zheng , Y. R. Du , G. Jiang , G. L. Xu , H. Wang , Cell Res. 2021, 31, 94. 32355286 10.1038/s41422-020-0317-6 PMC7853133

[10] B. Yao , Y. Cheng , Z. Wang , Y. Li , L. Chen , L. Huang , W. Zhang , D. Chen , H. Wu , B. Tang , P. Jin , Nat. Commun. 2017, 8, 1122. 29066820 10.1038/s41467-017-01195-y PMC5654764

[11] C. L. Xiao , S. Zhu , M. He , D. Chen , Q. Zhang , Y. Chen , G. Yu , J. Liu , S. Q. Xie , F. Luo , Z. Liang , D. P. Wang , X. C. Bo , X. F. Gu , K. Wang , G. R. Yan , Mol. Cell 2018, 71, 306. 30017583 10.1016/j.molcel.2018.06.015

[12] T. P. Wu , T. Wang , M. G. Seetin , Y. Lai , S. Zhu , K. Lin , Y. Liu , S. D. Byrum , S. G. Mackintosh , M. Zhong , A. Tackett , G. Wang , L. S. Hon , G. Fang , J. A. Swenberg , A. Z. Xiao , Nature 2016, 532, 329. 27027282 10.1038/nature17640 PMC4977844

[13] Y. Wang , X. Chen , Y. Sheng , Y. Liu , S. Gao , Nucleic Acids Res. 2017, 45, 11594. 29036602 10.1093/nar/gkx883 PMC5714169

[14] Z. Li , S. Zhao , R. V. Nelakanti , K. Lin , T. P. Wu , M. H. Alderman , C. Guo , P. Wang , M. Zhang , W. Min , Z. Jiang , Y. Wang , H. Li , A. Z. Xiao , Nature 2020, 583, 625. 32669713 10.1038/s41586-020-2500-9 PMC8596487

[15] C. Ma , R. Niu , T. Huang , L. W. Shao , Y. Peng , W. Ding , Y. Wang , G. Jia , C. He , C. Y. Li , A. He , Y. Liu , Nat. Cell Biol. 2019, 21, 319. 30510156 10.1038/s41556-018-0238-5

[16] J. Xiong , T. T. Ye , C. J. Ma , Q. Y. Cheng , B. F. Yuan , Y. Q. Feng , Nucleic Acids Res. 2019, 47, 1268. 30517733 10.1093/nar/gky1218 PMC6379677

[17] C. Lyu , Y. Niu , W. Lai , Y. Wang , Y. Wang , P. Dai , C. Ma , S. Chen , Y. Li , G. Jiang , Z. Liang , W. Ma , Z. Gao , W. Tong , H. Wang , Cell Discov. 2022, 8, 39. 35501312 10.1038/s41421-022-00399-x PMC9061847

[18] Y. Kong , L. Cao , G. Deikus , Y. Fan , E. A. Mead , W. Lai , Y. Zhang , R. Yong , R. Sebra , H. Wang , X. S. Zhang , G. Fang , Science 2022, 375, 515. 35113693 10.1126/science.abe7489 PMC9382770

[19] S. M. Kweon , Y. Chen , E. Moon , K. Kvederaviciutė , S. Klimasauskas , D. E. Feldman , Mol. Cell 2019, 74, 1138. 30982744 10.1016/j.molcel.2019.03.018 PMC6591016

[20] M. U. Musheev , A. Baumgärtner , L. Krebs , C. Niehrs , Nat. Chem. Biol. 2020, 16, 630. 32203414 10.1038/s41589-020-0504-2

[21] S. Chen , W. Lai , Y. Li , Y. Liu , J. Jiang , X. Li , G. Jiang , H. Wang , EMBO J. 2023, 42, 113684. 10.15252/embj.2023113684 PMC10390868 37366109

[22] Febrimarsa , S. G. G. , S. N. Barreira , M. Salinas‐Saavedra , C. E. Schnitzler , A. D. Baxevanis , U. Frank , EMBO J. 2023, 42, 112934. 10.15252/embj.2022112934 PMC10390872 37708295

[23] B. Liu , X. Liu , W. Lai , H. Wang , Anal. Chem. 2017, 89, 6202. 28471639 10.1021/acs.analchem.7b01152

[24] Y. Dai , B. F. Yuan , Y. Q. Feng , RSC Chem Biol 2021, 2, 1096. 34458826 10.1039/d1cb00022e PMC8341653

[25] S. A. Maier , J. R. Galellis , H. E. McDermid , J. Mol. Evol. 2005, 61, 776. 16245011 10.1007/s00239-005-0046-y

[26] M. Schinkmanová , I. Votruba , A. Holý , Biochem. Pharmacol. 2006, 71, 1370. 16513094 10.1016/j.bcp.2006.01.013

[27] M. Chen , M. J. Urs , I. Sánchez‐González , M. A. Olayioye , M. Herde , C. P. Witte , Plant Cell 2018, 30, 1511. 29884623 10.1105/tpc.18.00236 PMC6096584

[28] D. Bhaumik , J. Medin , K. Gathy , M. S. Coleman , J. Biol. Chem. 1993, 268, 5464. 8449909

[29] B. Wu , D. Zhang , H. Nie , S. Shen , Y. Li , S. Li , RNA Biol 2019, 16, 1504. 31318636 10.1080/15476286.2019.1642712 PMC6779375

[30] W. Niu , Q. Shu , Z. Chen , S. Mathews , E. Di Cera , C. Frieden , J. Phys. Chem. B 2010, 114, 16156. 20815357 10.1021/jp106041v PMC3005954

[31] J. Palis , Front Physiol 2014, 5, 3. 24478716 10.3389/fphys.2014.00003 PMC3904103

[32] C. Friend , W. Scher , J. G. Holland , T. Sato , Proc Natl Acad Sci U S A 1971, 68, 378. 5277089 10.1073/pnas.68.2.378 PMC388942

[33] A. S. Tsiftsoglou , I. S. Pappas , I. S. Vizirianakis , Pharmacol. Ther. 2003, 100, 257. 14652113 10.1016/j.pharmthera.2003.09.002

[34] E. Dzierzak , S. Philipsen , Perspect. Med. 2013, 3, a011601. 10.1101/cshperspect.a011601 PMC3684002 23545573

[35] E. R. Scaletti , K. S. Vallin , L. Bräutigam , A. Sarno , U. W. Berglund , T. Helleday , P. Stenmark , A. S. Jemth , J. Biol. Chem. 2020, 295, 4761. 32144205 10.1074/jbc.RA120.012636 PMC7152754

[36] L. N. Booth , A. Brunet , Mol. Cell 2016, 62, 728. 27259204 10.1016/j.molcel.2016.05.013 PMC4917370

[37] A. Unnikrishnan , W. M. Freeman , J. Jackson , J. D. Wren , H. Porter , A. Richardson , Pharmacol. Ther. 2019, 195, 172. 30419258 10.1016/j.pharmthera.2018.11.001 PMC6397707

[38] M. Cortés‐López , M. R. Gruner , D. A. Cooper , H. N. Gruner , A. I. Voda , A. M. van der Linden , P. Miura , BMC Genomics 2018, 19, 1. 29298683 10.1186/s12864-017-4386-y PMC5753478

[39] I. Shchukina , J. Bagaitkar , O. Shpynov , E. Loginicheva , S. Porter , D. A. Mogilenko , E. Wolin , P. Collins , G. Demidov , M. Artomov , K. Zaitsev , S. Sidorov , C. Camell , M. Bambouskova , L. Arthur , A. Swain , A. Panteleeva , A. Dievskii , E. Kurbatsky , P. Tsurinov , R. Chernyatchik , V. D. Dixit , M. Jovanovic , S. A. Stewart , M. J. Daly , S. Dmitriev , E. M. Oltz , M. N. Artyomov , Nat Aging 2021, 1, 124. 34796338 10.1038/s43587-020-00002-6 PMC8597198

