# Supplementary material for: Adenosine Deaminase‐Like Gene‐Carried Lentivirus Toolkit for Identification of DNA N6‐Methyladenine Origins
Source: Adv Sci (Weinh). 2024 Jul 18;11(35):2403376. doi: 10.1002/advs.202403376 (PMC11425204; doi:10.1002/advs.202403376)
Supplement: Supplementary file 1 — Supporting Information [file ADVS-11-2403376-s001.docx]

**Supplemental information**

**Adenosine deaminase-like gene-carried lentivirus toolkit for identification of DNA N^6^-methyladenine origins**

Ziyu Liang^[a, b]^, Shaokun Chen^[a]^, Yao Li^[a, b]^, Weiyi Lai^*[a]^, Hailin Wang^*[a, b, c, d]^

^[a]^The State Key Laboratory of Environmental Chemistry and Ecotoxicology, Research Center for Eco-Environmental Sciences, Chinese Academy of Sciences, Beijing 10085, China

^[b]^University of Chinese Academy of Sciences, Beijing 10049, China

^[c]^School of Environment and Health, Jianghan University, Wuhan 430056, China

^[d]^Hangzhou Institute for Advanced Study, University of Chinese Academy of Sciences, Hangzhou 310024, China

**Correspondence:** **[hlwang@rcees.ac.cn](mailto:hlwang@rcees.ac.cn) & wylai@rcees.ac.cn**

**Table of Contents**

[Methods	2](#_Toc161498258)

[Chemicals and Materials	2](#_Toc161498259)

[Animals	3](#_Toc161498260)

[Protein purification	4](#_Toc161498261)

[Enzyme activity analysis	5](#_Toc161498262)

[Cell culture and stable isotope tracer labelling	5](#_Toc161498263)

[Virus production	6](#_Toc161498264)

[Transfection of mammalian cells	7](#_Toc161498265)

[Western blotting analysis	8](#_Toc161498266)

[Differentiation of MEL cells	8](#_Toc161498267)

[Purification of bone marrow cells and flow cytometry analysis	9](#_Toc161498268)

[Genomic DNA extraction and enzymatic digestion	9](#_Toc161498269)

[UHPLC-MS/MS analysis	9](#_Toc161498270)

[Statistical analysis	10](#_Toc161498271)

[Supporting Figures	11](#_Toc161498272)

[Supporting Table	16](#_Toc161498273)

[Supporting Reference	17](#_Toc161498274)

# Methods

## Chemicals and Materials

N^6^-methyl-2’-deoxyadenosine (6mdA), 2’-deoxyadenosine (dA), N^6^-methyladenosine 5'-monophosphate (m6rAMP) were ordered from Sigma-Aldrich (St. Louis, MO, USA). 2’-deoxycytidine (dC) were purchased from J&K Scientific (Beijing, China). [^15^N_5_]-adenosine ([^15^N_5_]-rA), [^15^N_3_]-deoxycytidine ([^15^N_3_]-dC), and [D_3_]-L-methionine were ordered from Cambridge Isotope Laboratories Inc. (Andover, MA, USA). N^6^-methyladenosine (m6rA), [D_3_]-N^6^-methyladenosine ([D_3_]-m6rA), [D_3_]-N^6^-methyl-2’-deoxyadenosine ([D_3_]-6mdA) was ordered from Toronto Research Chemicals Inc. (North York, Canada). [^15^N_4_]-deoxyinosine ([^15^N_4_]-dI), and [^15^N_4_]-inosine ([^15^N_4_]-rI) were synthesized in our own lab. phosphodiesterase I (SVP) were ordered from Sigma-Aldrich Inc. (St. Louis, MO, USA). SuperNuclease was ordered from Sino Biological Inc. (Beijing, China). Calf intestinal alkaline phosphatase (CIP) was ordered from New England Biolabs Inc. (Ipswich, MA, USA). The DYKDDDDK tag Rabbit Polyclonal Antibody was purchased from Protein Tech (Rosemont, IL, USA). The HA tag Rabbit Polyclonal Antibody and GAPDH Polyclonal Antibody were purchased from Proteintech. The secondary antibodies goat anti-rabbit IgG (H+L) DyLight 800 was purchased from Bioworld Technology, Inc. (St. Louis Park, MN, USA). Anti-mouse Ter-119 (PE) and anti-mouse CD71(PE-CY7) for flow cytometry analysis were purchased from Biolegend (San Diego, USA).

## Animals

C57BL/6J male mice of different ages were ordered from Vitalriver Company (Beijing, China). All animal procedures were approved by and performed according to the guidelines of the Animal Ethics and Welfare Committee of Research Center for Eco-Environment Sciences, Chinese Academy of Science, approval No. AEWC-RCEES-2021049.

## Protein purification

For cloning of Adal, total RNA was extracted from mES cells and reverse-transcribed into cDNA using HiScript® III 1st Strand cDNA Synthesis Kit (Vazyme, China). The full length of cDNA fragment encoding *Adal* was amplified then inserted into pMAL-c5x. The MBP tag was attached at protein amino terminus. The pMAL-c5x-MBP-*Adal* plasmid was transformed into trans5α competent cells (TransGen Biotech, China). After incubation at 37 ^°^C overnight, monoclones were picked for proper insert identification by Sanger sequencing. Adal mutant H207A was constructed using M5 Site-Directed Mutagenesis Kit (Mei5 Biotechnology, China). Primers for construction of pMAL-c5x-MBP plasmids and point mutation are listed in the Table S1.

For protein purification, all plasmids were transformed into Dam KO BL21 (DE3) competent cells, ^[1]^ then proliferated in LB liquid medium until the OD reached 0.4-0.6. Protein expression was induced by adding IPTG to a final concentration of 0.2 mM. The cells were incubated at 14 ^°^C for another 24 hours. All proteins were purified using a self-packing hydrophobic interaction chromatography column and a prepacked MBP-Trap HP 5 mL column (GE Healthcare, Uppsala, Sweden). Purified MBP-Adal and MBP-A Adal H207A protein were heated at 95 ^°^C for 10 minutes and identified by SDS-PAGE analysis. The quantification was achieved with a BCA Protein Assay Kit (Beyotime, China).

## Enzyme activity analysis

To prepare the substrates for enzymatic reaction in vitro, 6mdAMP and m6rAMP were needed. 6mdAMP was acquired by digesting 6mdA-rich plasmid DNA. 2.5 μg plasmids were digested using 1 U of SuperNuclease, and 0.02 U of SVP in a 100 μl reaction mixture containing 10 mM Tris-HCl (pH 8.0), 2 mM Mg^2+^ for 6 hours. The mixture was filtered by passing through an ultrafiltration tube (MW cutoff: 3 kDa; Pall Corporation, USA). To prepare substrate stock solution, above filtrated solution was supplemented with m6rAMP at a final concentration of 600 nM. The enzymatic reaction was conducted at 37 ^°^C for 1 hour in a 50 μL reaction mixture containing 20 mM Tris-HCl (pH 7.0), 0.1 μg/μl BSA, 2 mM DTT, 1 μL of the substrate stock solution and 2 μg of Adal or Adal mutant. We set up a group with thermal-denatured protein as negative control. Reaction products were treated with proteinase K (New England Biolabs, USA) at 55 ^°^C for 30 min, and heated at 95 ^°^C for 10 min to inactive enzyme. All samples were incubated with 5.0 U CIP for another 1 hour followed by passing through ultrafiltration tubes. m6rAMP, 6mdAMP, rIMP and dIMP were detected in the form of m6rA, 6mdA, rI, and dI. The amount of nucleoside was quantified using UHPLC-MS/MS.

## Cell culture and stable isotope tracer labelling

mES cells (129 SvEv) were seeded in 0.1% sterilized gelatin coated dishes and cultured in DMEM high glucose medium (Thermo Fisher Scientific, USA) supplemented with 0.1 mM nonessential amino acids (Gibco, USA), 0.1 mM β- mercaptoethanol, 1.0 mM sodium pyruvate (Gibico, USA), 1.0 μM PD0325901 (Stemgent, Cambridge, USA), 2.0 mM L-Glutamine (Gibco, USA), 1000 U/mL mouse leukemia inhibitor factor (mLIF) (Millipore, Billerica, USA), 3.0 μM Stemolecule CHIR99021 (Stemgent, Cambridge, USA), 0.35% penicillin-streptomycin solution (Thermo Fisher Scientific, USA), and 20% fetal bovine serum (FBS) (Thermo Fisher Scientific, USA).

HEK-293T, C2C12, and NIH3T3 cells were cultured in DMEM high glucose medium supplemented with 10% FBS, 1% penicillin-streptomycin solution. MEL cells were cultured in RPMI-1640 (Thermo Fisher Scientific Inc., USA) medium supplemented with 15% FBS, 1% penicillin-streptomycin solution. Of note, all the cell lines were cultured in a humidified incubator at 37 ^°^C supplemented with 5% CO_2_.

For stable isotope tracer labelling, 2.0 ×10^5^ HEK-293T, 1.0 ×10^5^ mES, NIH3T3 and C2C12 cells were plated in 6-well cell, cultured in 2.0 mL medium. The final concentration of [^15^N_5_]-rA and [D_3_]-L-methionine was 20 μM and 30 μg/mL, respectively. The medium was replaced with fresh medium containing stable isotope every 24 h, or as stated.

## Virus production

*Adal* and mutant H207A CDS fragments were prepared as stated above, then inserted into pLVX-EF1α-IRES-puro vector for overexpression experiments. Primers for lentivirus overexpression plasmids construction are listed in the Table S1. For virus production, 4.3 µg of pLVX-EF1α-IRES-puro, 6.5 µg of pMDLg/pRRE, 3.7 µg of pMD2.G and 2.8 µg of pRSV-Rev were diluted with 3 mL Opti-MEM™ (Gibco, USA) medium, then transfected with Lipofectamine™ 3000 (Thermo Fisher Scientific, USA) into HEK-293T cells. 6 hours post transfection the media was replaced with 10 mL fresh HEK-293T cultured medium as stated. Viral supernatants were collected every 24 hours for a total of two collections, and filtered with a 0.45 μm filter (PALL, USA). Virus solution was concentrated with 4× PEG8000-NaCl (20% PEG8000 (w/v), 0.6 M NaCl) solution for 24 hours at 4 ^°^C, then was centrifuged at 4000 rpm for 30 minutes at 4 ℃. Virus was stored at −80 ^°^C.

## Transfection of mammalian cells

For lentiviral transfection, 1.0 ×10^5^ HEK-293T cells or 5.0 ×10^4^ mES, NIH3T3 and C2C12 cells were plated in 12-well cell culture cluster with 500 μL medium. Virus was added with Viral infection reagents HitransG P (Genechem, China). After 16 h, the transduction solution was replaced by 1 mL fresh cultured medium. Stably transfected cells were selected in the presence of 1 µg/mL puromycin after 24 h. MEL and cells were plated at 1.0 ×10^5^ cells/mL in 12-well cell, followed by centrifuging with virus and HitransG P at 3000rpm for 1.5 h at RT. After 16 h, the transduction solution was replaced by 1 mL fresh cultured medium. Stably transfected cells were selected in the presence of 0.5 µg/mL puromycin after 48 h.

For transient transfection of pcDNA-Dam V181N 3×HA, 3.0 ×10^5^ HEK-293T cells were plated in 6-well cell culture cluster with 1 mL medium. Plasmids were mixed with PEI at ratio 1: 3 before adding in medium. Another 1 mL fresh medium was added after 24 h.

## Western blotting analysis

Total protein of cultured cells was extracted with RIPA lysis buffer (Beyotime, China) containing a protease inhibitor (Thermo Fischer Scientific, USA) and a phosphatase inhibitor (Nacalai Tesque, Chnia). The concentration of protein was measured by BCA Protein Assay Kit. Heat-inactivated protein samples were separated on 4–12% NUPAGE Bis-Tris gels (Thermo Fisher Scientific, USA) in MOPS buffer (Thermo Fisher Scientific, MA, USA), and subsequently transferred to polyvinylidene difluoride membranes (Merck Millipore, Germany). Membranes were blocked with 5.0% skimmed milk powder (BD Biosciences, USA) agent for 1 h, then incubated with diluted (1:1000) primary antibodies overnight at 4 ^°^C. After washed with Tris-buffered saline (Sangon Biotech, China) containing 0.1% Tween-20 (Solarbio, China), membranes were incubated with appropriately diluted (1:1500) Goat Anti-Rabbit IgG(H+L) Dylight 800 (Bioworld, USA) for another 1 h at room temperature. Finally, the target protein bands were detected with Odyssey Infrared Imaging System (Li-COR Biosciences, USA). The target band intensities were quantified and analyzed by Image J. Each western blot was normalized using consistent internal control, GAPDH. Three normalized values for each protein were used for calculating average and standard deviation.

## Differentiation of MEL cells

For cell differentiation, 7.5 ×10^5^ MEL cells were plated in T25 tissue culture flasks with 15 mL of medium supplemented with 1.2% DMSO (Coolaber, China). 30 μg/mL [D_3_]-L-methionine was added at the beginning of cell differentiation, which was supplied every 24 hours in the following 4 days.

## Purification of bone marrow cells and flow cytometry analysis

For bone marrow cells collection, C57BL/6J male mouse was euthanized under sterile conditions. The hind legs were removed, then muscles and fibrous tissue from the bones were cut off with forceps and scissors. After Trimming both ends of bones, PBS buffer was drawn with a 2 mL syringe to flush out the marrow into a 15 mL centrifuge tube. The solution was filtered by 30 μm Pre-Separation Filters (Miltenyi Biotec, Germany), and centrifuged at 300 × g for 5 minutes at 4 °C. Isolated BMCs were incubated with PE anti-mouse Ter-119 (Biolegend, USA) and PE-CY7 anti-mouse CD71 (Biolegend, USA) for 15 min on ice in PBS buffer and subjected to flow cytometry on BD Aria II flow cytometer (BD Biosciences, USA) to obtain erythroblasts (Ter119+ CD71+) and Ter119- cells.

## Genomic DNA extraction and enzymatic digestion

The genomic DNA was extracted using the manufacturers of the Promega wizard® genomic DNA purification kit (Promega, WI, USA). To obtain deoxyribonucleosides, per 5.0 µg DNA was digested with 1 U SuperNuclease, 0.5 U CIP, and 0.25 U SVP at 37 ^°^C overnight. Ultrafiltration tubes were used to remove enzymes. All ultrafiltered samples were then analyzed by UHPLC -MS/MS.

## UHPLC-MS/MS analysis

The UHPLC-MS/MS analysis was performed on an Agilent 1290 series UHPLC system coupled with an ESI-triple quadrupole mass spectrometer (6495, Agilent Technologies, USA). The mass spectrometer was operated under positive ionization using multiple reactions monitoring (MRM) mode. The selective MRM transitions were monitored as follows: *m/z* 266→150 for 6mdA, *m/z* 269→153 for [D_3_]-6mdA, *m/z* 270→154 for [^15^N_4_]-6mdA, *m/z* 271→151 for [^15^N_5_]-6mdA, *m/z* 282→150 for m6rA, *m/z* 285→153 for [D_3_]-m6rA, *m/z* 253→137 for dI, *m/z* 257→141 for [^15^N_4_]-dI, *m/z* 269→137 for rI, *m/z* 273→141 for [^15^N_4_]-rI, *m/z* 268→136 for rA, *m/z* 272→140 for [^15^N_4_]-rA, *m/z* 273→141 for [^15^N_5_]-rA, *m/z* 228→112 for dC, *m/z* 231→115 for [^15^N_3_]-dC.

## Statistical analysis

Statistical significance was assessed using paired two-tailed Student's t -tests. Data was presented as mean ± SD. *p* < 0.05 was considered to indicate statistical significance.

# Supporting Figures

**Figure S1.** Sequence and protein information of Adal mutant H207A. (a) Sequence alignment of the Zn^2+^ binding site residues among human (Hs), bovine (Bt), mouse (Mm), Xenopus laevis (XI), zebrafish (Dr), Arabidopsis thaliana (At), Drosophila melanogaster (Dm), Caenorhabditis elegans (Ce) ADAL and human, mouse adenosine deaminase (ADA). The Zn^2+^ binding sites are numbered underneath according to mouse Adal (His23, His25, His207, Asp292). (b) Base alteration of Adal mutant H207A. (c) SDS-PAGE analysis of purified MBP-Adal WT and mutant H207A.

**Figure S2.** UHPLC-MS/MS chromatograms of substrates (6mdAMP (a) and m6AMP (c)) and products (dIMP (b) and rIMP (d)) upon deamination reaction mediated by purified MBP-Adal WT and mutant H207A. 6mdAMP, m6rAMP, dIMP and IMP were detected in the form of dephosphorylated products.

**Figure S3.** Sanger sequencing of FLAG-mouse *Adal* WT Sequence (a) and FLAG-mouse *Adal* H207A Sequence (b).

**Figure S4.** Biosynthetic pathways of adenosine methylase-caused bona fide DNA 6mdA (pr6mdA) and misincorporated DNA 6mdA (i6mdA) production. The black arrows indicate the relevant paths of pr6mdA production. The red arrows indicate the relevant paths of i6mdA production. Adal, Ak1, and Mth1 were reported as key proteins that participated in regulating the generation of i6mdA. m6rAMP: N^6^-methyl-adenosine 5'-monophosphate; 6mdAMP: N^6^-methyl-2’-deoxyadenosine 5'-monophosphate; 6mdADP: N^6^-methyl-2’-deoxyadenosine 5'- diphosphate; 6mdATP: N^6^-methyl-2’-deoxyadenosine 5'- triphosphate; IMP: inosine 5’-monophosphate; dIMP: 2’-deoxyinosine 5’-monophosphate; SAM: S-Adenosylmethionine; SAH: S-adenosyl L-homocysteine; Ak1: adenylate kinase 1; Rnr: ribonucleotide reductases; Ndpk: nucleoside diphosphate kinase; Mth1: MutT homolog 1.

**Figure S5.** Statistical results of isotope labeling rates in C2C12, NIH3T3 and MEL cells. (a-b) The [^15^N_5_]-rA labeling rate of C2C12 (a) and NIH3T3 (b) cells was verified by the percent of [^15^N_4_]-rA and [^15^N_5_]-rA. (c) The [D_3_]-Met labeling rate of MEL cells was verified by the percent of [D_3_]-m6rA. Error bars are S.D. (n = 3 biological replicates).

# Supporting Table

**Table S1 Primer sequence for PCR**

| **Primers for point mutation** | |
| --- | --- |
| **H207A-F** | 5'-TGAAGTTGGCATTGGCTCTTGCCGAGA-3' |
| **H207A-R** | 5'-GCCAATGCCAACTTCAGACCTGCTTTTT-3' |
| **Primers for pMAL-c5x-MBP plasmids construction** | |
| **MBP-Adal WT/H207A-F** | 5'-TCCGGTAGTGGAGGACCAGGGTCCATGGAGGCCGGACAGCAGTGGC-3' |
| **MBP-Adal WT/H207A-R** | 5'-TGGTGGTGGTGGTGGTGCTCGAGTCAAAAGTAGTTCACCTCATTA-3' |
| **Primers for lentivirus overexpression plasmids construction** | |
| **FLAG-Adal WT/H207A-F** | 5'-ACGACGATGACAAGACTAGTATGGAGGCCGGACAG-3' |
| **FLAG-Adal WT/H207A-R** | 5'-CCGCGGCCGCTCTAGTCAAAAGTAGTTCACCTCATTACAG-3' |

# Supporting Reference

[1] Z. Chen, Y. Liu, H. Wang, *Biosci. Rep*. 2021, 41, 3.
